# Graceful gait: virtual ballet classes improve mobility and reduce falls more than wellness classes for older women

**DOI:** 10.3389/fnagi.2024.1289368

**Published:** 2024-01-24

**Authors:** Elinor C. Harrison, Allison M. Haussler, Lauren E. Tueth, Sidney T. Baudendistel, Gammon M. Earhart

**Affiliations:** ^1^Program in Dance, Performing Arts Department, Washington University in St. Louis, St. Louis, MO, United States; ^2^Program in Physical Therapy, Washington University in School of Medicine St. Louis, St. Louis, MO, United States; ^3^Department of Neurology, Washington University School of Medicine in St. Louis, St. Louis, MO, United States; ^4^Department of Neuroscience, Washington University School of Medicine in St. Louis, St. Louis, MO, United States

**Keywords:** dance, mobility, kinesthetic empathy, dual task, socialization, ballet

## Abstract

**Introduction:**

Dance is an effective and motivating form of exercise for older women, but few studies have quantified the benefits of virtual dance classes nor, specifically, ballet. This study tested the effectiveness of virtual ballet compared to virtual wellness classes, with the goal of reaching underserved populations. It is among the first to explore the effects of virtual classical ballet on functional gait mobility, balance, and quality of life measures in older women.

**Methods:**

Older women were recruited in two waves and randomized to two groups: a ballet class modified for older adults and a wellness-based control class. Both groups received 12 weeks of online classes, meeting twice per week for 45-min sessions. Classes were taught by a local company that offers community-based ballet classes. The same instructor led both the ballet and the wellness classes. Pre- and post-intervention assessments include gait and balance testing using wearable inertial sensors and self-report outcomes including quality of life and mood questionnaires.

**Results:**

Forty-four older women completed the study: Ballet group (*n* = 21, 67.81 ± 7.3 years); Wellness group (*n* = 23, 69.96 ± 6.7 years). Pre- to post-intervention, both groups increased velocity on the two-minute walk test (*F*_1,42_ = 25.36, *p* < 0.001) and improved their time on the Timed Up and Go (*F*_1,42_ = 4.744, *p* = 0.035). Both groups improved balance on the Mini-BESTest (*F*_1,42_ = 38.154, *p* < 0.001), increased their scores on the Activities-Specific Balance Confidence Scale (*F*_1,42_ = 10.688, *p* < 0.001), and increased quality of life via the Short Form Health Survey (*F*_1,42_ = 7.663, *p* = 0.008). The ballet group improved gait variability in the backward direction (*F*_1,42_ = 14.577, *p* < 0.001) and reduced fall rates more than the wellness group [χ^2^(1) = 5.096, *p* = 0.024].

**Discussion:**

Both virtual ballet and wellness classes improve select measures of gait, balance, and quality of life. The benefits seen in both groups highlight the importance of considering social interaction as a key component when developing future interventions to target mobility in older women.

## 1 Introduction

Falls are a major cause of disability in older adults. Every second of every day an older adult, aged 65 or older, suffers a fall in the US, leading to 30 million falls each year (CDC, 2019). Women, who are more likely than men to experience bone density loss in older age, are particularly susceptible to falls and account for 75% of all hip fractures regardless of gender (Gale et al., 2016). Even a single fall can be debilitating, leading to a fear of falling, withdrawal from activities, and reduced quality of life (Bower et al., 2016).

The two primary factors that contribute to fall risk among older adults are decline in gait and decline in balance ability (Kyrdalen et al., 2019). Gait impairment affects one third of the population over 70 years of age and represents a major cause of falls (Verghese, 2006). Gait speed, in particular, is a robust marker of overall health, as reductions in self-selected gait speed with aging can predict adverse events, future disability, healthcare utilization, and even mortality (Cesari et al., 2005). Slower walking can also lead to more variability between steps, rendering walking less stable (Kang and Dingwell, 2008). Increased gait variability in older adults is correlated to higher fall risk (Hausdorff et al., 2001). Multifactorial interventions for older adults that increase gait speed and decrease gait variability may help reduce fall rates (Gillespie et al., 2021).

Balance is a complex activity that requires coordinating multiple body systems. Effective balance involves maintaining upright posture during static conditions as well as facilitating movement during dynamic tasks, such as walking (Horak, 2006). Balance decline in aging involves reduced strength, flexibility, and sensory loss, which collectively contribute to fall risk (Kanekar and Aruin, 2014). Older adults at risk of falling, when compared to those who are not, are unable to hold static postures as long (Araujo et al., 2022), exhibit increased postural sway during standing balance tasks (Oliveira et al., 2018), and perform worse on dual cognitive-motor tasks (Verghese et al., 2002).

Dance is a multi-modal artform that provides an effective therapy for addressing gait and balance impairments in older adults as it combines multi-sensory elements including proprioceptive, visual, auditory, and motor control techniques (Earhart, 2009). In-person dance interventions for older adults frequently show benefits to functional mobility (Hwang and Braun, 2015; Britten et al., 2017). Gait improvements have been reported across a broad range of dance styles showing improvements in stride velocity (Granacher et al., 2012), walking endurance (Shigematsu et al., 2002; Hui et al., 2009), and muscle function (Cepeda et al., 2015). Balance improvements are reported in a similarly wide variety of dance styles including contemporary (Ferrufino et al., 2011; Coubard et al., 2014), jazz (Wallmann et al., 2009), ballroom (Cepeda et al., 2015), salsa (Granacher et al., 2012), tango (Hackney et al., 2007; Hackney and Earhart, 2009; Earhart, 2013; Sofianidis et al., 2017), and traditional folk dance (Eyigor et al., 2009; Sofianidis et al., 2009; Pacheco et al., 2016). Furthermore, cross-sectional studies show that older adults who dance regularly have better balance capabilities than those who do not (Verghese, 2006; Dewhurst et al., 2015).

Classical ballet interventions for older adults, however, remain underrepresented in the literature despite ample evidence to suggest that they might be useful in older populations (Hwang and Braun, 2015; Weighart and DiPasquale, 2020). Ballet trains postural stability through a mix of static and dynamic processes that may translate to gait and balance improvements. The use of a ballet barre renders training feasible and safe for older adults, while encouraging stability and upright posture during standing exercises. Ballet is highly adaptable to home studio settings, as stationary chairs readily substitute for ballet barres and much of the class period can be done in a small room with minimal shifting of the camera. Ballet classes emphasize sensorimotor integration (Tanabe et al., 2014) and teach specific visual and proprioceptive techniques that may aid postural control and body awareness (de Mello et al., 2017). Ballet combinations are cognitively challenging and require complex coordination of multiple body parts. Emphasis on movement sequencing, timing, and efficiency may help with dynamic movement tasks (Kiefer et al., 2011). Ballet may also teach strategies for complex movement tasks, such as backward or dual task walking, that frequently lead to falls (Muir-Hunter and Wittwer, 2016; Bayot et al., 2020). Focusing on full-body stabilization during ballet may challenge the body to constantly recalibrate and adjust to destabilizing forces (Weighart and DiPasquale, 2020).

Dance interventions not only address mobility issues, but also promote cognition, emotional expression, kinesthetic awareness, and social engagement (Hwang and Braun, 2015; Clifford et al., 2023). During the COVID-19 pandemic, many dance therapy classes moved online, which afforded the convenience and safety of participating from one’s own home but removed elements of social interaction and group support that are naturally structured into in-person classes (Bek et al., 2022). Kinesthetic empathy, or the ability to perceive others’ emotions through action observation, is a key component of in-person classes (Christopher and Tamplin, 2022) that may occur less spontaneously during virtual classes. While in-person classes have resumed, online classes often remain available for people who are unable to travel or need flexibility to participate from home; however, little is known about how much benefit online classes provide.

Though the main objective of the interventions was to improve functional mobility, we also assessed the synergistic interactions of mind and body reflected through complex motor dual-tasks, quality of life, and mood. In order to test the effects of ballet on gait and balance, he primary outcome measures were gait velocity and postural stability. Secondary measures were balance confidence, fall frequency, quality of life, and mood measures. We hypothesized that the ballet intervention would be more effective at improving gait and balance measures than the wellness intervention, but that both interventions would equally improve quality of life and mood.

## 2 Methods

The interventions consisted of a ballet class that utilized a pre-existing model of classical ballet modified for older adults, and a wellness class that served as a control intervention. The original intent of the study was to research in-person ballet classes, but due to COVID-19, the study moved entirely online well before the start of the study. The study was approved by the Washington University IRB and all participants gave written informed consent.

### 2.1 Participants

Older women aged 55 and above were recruited from local community centers, senior residences, and senior assistance organizations. Participants were recruited via flyers in two waves. Covariate adaptive randomization (Kang et al., 2008) counterbalancing for age was used to divide participants into two groups: a ballet class and a wellness class.

Recruitment was limited to women as they are at higher risk of frailty, hip fractures from falls, and physical inactivity than men (CDC, 2019). Additionally, women report higher anxiety and more depressive symptoms than men (Nair et al., 2021) and may therefore benefit more from interventions targeted at improving quality of life and mood.

In addition to the inclusion criteria of age and gender identity, participants needed to be able to walk independently with or without an assistive device for at least 5 min. Exclusion criteria were ballet training in the last 2 years; evidence of dementia (Mini-Mental Status Examination <24); language, visual, or hearing barriers to participation; and/or history of orthopedic or other medical problems that limit ability to participate safely in the intervention. To be included in the final analysis, participants needed to attend 70% (minimum 17 of 24) of the classes.

Sixty-five participants were recruited but 21 dropped out or had poor attendance due to extenuating factors during the pandemic including health reasons, caretaking responsibilities, and technology issues. Several people randomized to the wellness class dropped out before the classes started because they were displeased with their class placement. Attendance was taken daily; participants missed classes due to a range of conflicts such as work obligations, caretaking responsibilities, technology issues with Zoom, and vacations. Forty-four participants completed the intervention with enough classes to be included in the final analyses: ballet group (*n* = 21); wellness group (*n* = 23) (Table 1). The groups were well-matched in terms of baseline dance experience and mobility measured via gait speed. We made concerted effort to include participants from under-represented groups.; 20.45% (9/44) of the participants were non-white.

**Table 1:** Participant demographics.

		Ballet	Wellness	*p*
N		21	23	
Age		67.81 (7.3)	69.96 (6.7)	*0.33*
Race (*n*)	White	16	19	
	Black	4	3	
	Asian	1	1	
Comorbidities (*n*)	0	5	3	
	1	6	10	
	2	7	7	
	>2	3	3	
Retirement status		57%	83%	
Education level	Some college or trade school	5%	22%	
	College degree (Associates)	5%	17%	
	College degree (Bachelors)	47%	13%	
	Post college	43%	48%	
Living situation	Alone	28%	22%	
	With partner	48%	61%	
	With family (in addition to partner)	0%	4%	
	With family (other than partner)	24%	13%	
Dance experience (years)		3.26 (7.1)	3.05 (3.4)	*0.14*
Baseline velocity (m/s)		1.40 (0.28)	1.49 (0.25)	*0.29*
Baseline Mini-BESTest, median (range)		24 (13,32)	24 (10,31)	*0.1*

Values are mean ± SD unless noted. *T*-tests were used for between-group comparisons. Baseline velocity is taken from 2MWT. Baseline Mini-BESTest score refers to overall score.

### 2.2 Class structure

All classes were taught by a local organization, *Vitality in Motion* https://vitalityinmotion.com/, that offers high-quality, community-based ballet classes. Both groups received 12 weeks of online classes, in accordance with dosage recommendations for dance interventions (Hackney et al., 2007; Hackney and Earhart, 2009, 2010; Hwang and Braun, 2015). Both groups met two mornings per week over Zoom (Zoom Video Communications Inc., San Jose CA) for 45-min sessions, as is the standard length of *Vitality in Motion* classes.

The ballet classes followed conventional class formats utilizing ballet movement vocabulary and were set to classical music (Table 2). Classes were tailored to older populations and modified according to individual or group needs. Each class began with seated warm-up exercises that progressed to standing “barre work” using chairs for support. Some “center work” done in the middle of the room without the barre allowed participants to practice movement sequencing, memorization of steps, and expressive choreography while freely moving in space.

**Table 2:** Structure of ballet and wellness classes.

Ballet class Greetings and announcements Opening warm-up *Stretching* *Mobility exercises* Ballet barre behind chair: *Pliés* *Tendus* *Dégagés* *Ronde de jambes* *Piqués* *Petite battements* *Adagio: développés/enveloppés/fondus* *Balancés* Ballet walking exercises in center Reverence	Wellness class Greetings and announcements Opening presentation on a health topic Example topics: *Isolation during Covid* *Nutrition* *Hydration* *Physical activity* *Mindfulness* *Sleeping* *Brain health* *Mental health* *Fall prevention* Discussion of topic among participants *Participants given equal time to speak* Closing remarks

Modifications for virtual settings were intended to closely replicate the in-person class experience. Students were able to see and mimic the teacher’s movements throughout the class. There were no mirrors, but participants used their own cameras to provide visual feedback of their own performance. The online format via Zoom allowed for constant monitoring of bodies synchronizing in space, allowing for embodied interaction with others in real-time. A member of the research team was present to monitor each session and help participants with technology needs and ensure safety. To account for social interactions that typically occur during group classes, the monitor opened up the Zoom class 10 min early for participants to unmute and chat. We had no issues with Zoom during the classes. Some issues for individual participants did arise (i.e., not being able to connect to wifi, not being able to hear, not being able to find adequate space) and resulted in participants not being included in analyses.

The control intervention was selected as an active intervention that held steady variables such as class size, duration, socialization, and instructor demeanor but did not involve movement. We thought this a better comparison than utilizing a control condition that involved no intervention, which is often the default in early phase studies of movement interventions. The wellness classes (Table 2) were led by the same instructor as the ballet classes to control for any effect of the instructor’s demeanor or personality. In these education-based classes, participants discussed topics related to wellness and aging. No movements or exercises were performed during any of the wellness classes. Educational presentations were given each class by the instructor and followed with time for the participants to discuss their own experiences. This control comparator intervention was selected based on recommendations by an NIH expert panel on health-related behavioral interventions (Freedland et al., 2019).

### 2.3 Assessments

All participants underwent a comprehensive evaluation at two time points: pre-intervention and post-intervention. Evaluations occurred within 2 weeks of the start/end of classes. The same battery of assessments was used at both time points. Assessors were blinded to group assignment. As falls are multifactorial, commonly used assessments of gait and balance do not adequately predict fall risk in older adults in isolation (Omaña et al., 2021); therefore, multiple measures were assessed in this study.

The assessments consisted of motor and self-reported outcomes. During the motor exam, gait was captured during free walking using six wearable sensors (feet, wrists, sternum, lumbar) (APDM Mobility Lab, APDM Inc., Portland, OR). Sensors provided detailed information regarding spatiotemporal features of gait during the 2-min walk test (2MWT) and the 10-m walk test (10MWT). The 10MWT was assessed in four conditions, each performed three times: comfortable pace, fast as possible pace, dual-task (while doing a verbal cognitive task), and backward walking. Variables of interest included: stride velocity, stride length, and stride time as well as the coefficient of variation (CV) of stride velocity, stride length, and stride time.

Balance was assessed via the Mini Balance Evaluation Systems Test (Mini-BESTest), a well-validated tool that measures both static and dynamic balance, functional mobility, and gait (Marques et al., 2016). Postural sway during static balance tasks was measured via wearable sensors during single leg stance (SLS) performed twice on each leg in 30-s bouts. Balance variables were selected based on high validity and reliability and calculated within APDM software. These included duration, total sway area (computed as the area included in the acceleration per unit of time), jerk (smoothness of sway from time derivative of acceleration), mean velocity, and mean RMS (root mean square of the ACC time series). Greater static balance control is associated with higher duration (Baker et al., 2021) and lower sway area (Sohn et al., 2023), jerk (Mancini et al., 2012), mean velocity (Borysiuk et al., 2018), and mean RMS (Alsubaie, 2020). Additionally, self-reported balance confidence was measured via the Activities-Specific Balance Confidence Scale (ABC) (Schepens et al., 2010).

Sensors captured three trials of both the simple Timed Up-and-Go (TUG) and dual-task cognitive TUG (DT-TUG) in which participants subtracted backwards from 100 by threes. Dual task cost was calculated according to the following formulaw:

$\left(DT\;\mathrm{cost}=\frac{DT\;\mathrm{TUG}\;\mathrm{duration}-\mathrm{TUG}\;\mathrm{duration}}{\left(\mathrm{TUG}\;\mathrm{duration}\right)}\times 100\right)$ (Piche et al., 2023).

Questionnaires assessed quality of life [Short Form Health Survey (SF-36)] and mood [Geriatric Depression Scale (GDS)]. Self-reported fall frequency was reported at both time points and summated for the 3 months prior. Participant demographic information was also collected. Most common comorbidities reported were arthritis, high cholesterol, and hypertension.

Statistical analyses were conducted using IBM SPSS 27. We employed two-way repeated measures (RM) ANOVAs with group (ballet vs. wellness) and time (pre- vs. post-intervention) as factors to determine how the interventions impacted outcome variables. Fall rates were compared via logistic regression and the likelihood ratio was calculated via a Chi-square test. Post-hoc pairwise comparisons were used as appropriate, and Bonferroni corrections were used to correct for multiple comparisons. Statistical significance was set at *p* = 0.05.

## 3 Results

### 3.1 Gait results

In the 2MWT, there were main effects of time for velocity and stride length, which increased from pretest to posttest for both groups (Table 3). There was also a main effect for stride time which decreased for both groups.

**Table 3:** Gait characteristics.

		**Ballet**		**Wellness**				
		Pre	Post	Pre	Post	Main effect of time	Main effect of group	Interaction effect
2 MWT	Velocity (m/s)	1.40 (0.27)	1.47 (0.29)	1.49 (0.26)	1.54 (0.27)	<0.001*	0.336	0.520
	Stride length (m)	1.35 (0.18)	1.36 (0.18)	1.36 (0.16)	1.38 (0.16)	0.002*	0.768	0.578
	Stride time (s)	0.97 (0.08)	0.94 (0.08)	0.92 (0.07)	0.91 (0.02)	<0.001*	0.070	0.131
	10 MWT comfortable	Velocity (m/s)	1.17 (0.23)	1.18 (0.20)	1.22 (0.16)	1.22 (0.21)	0.628	0.447	0.434
Stride Length (m)		1.22 (0.17)	1.23 (0.16)	1.24 (0.12)	1.24 (0.14)	0.542	0.765	0.961
Stride Time (s)		1.07 (0.08)	1.05 (0.06)	1.02 (0.07)	1.03 (0.07)	0.652	0.121	0.041*
10 MWT dual task		Velocity (m/s)	1.02 (0.23)	1.04 (0.23)	1.10 (0.17)	1.08 (0.21)	0.767	0.323	0.251
	Stride Length (m)	1.12 (0.18)	1.14 (0.17)	1.16 (0.13)	1.16 (0.14)	0.475	0.599	0.370
	Stride Time (s)	1.13 (0.11)	1.12 (0.10)	1.06 (0.08)	1.09 (0.11)	0.715	0.105	0.112
	10 MWT fast as possible	Velocity (m/s)	1.53 (0.27)	1.56 (0.27)	1.64 (0.22)	1.64 (0.21)	0.229	0.177	0.264
Stride length (m)		1.38 (0.16)	1.38 (0.16)	1.40 (0.15)	1.42 (0.15)	0.114	0.498	0.456
Stride Time (s)		0.91 (0.07)	0.90 (0.08)	0.86 (0.06)	0.87 (0.06)	0.699	0.058	0.108
10 MWT backward	Velocity (m/s)	0.80 (0.26)	0.83 (0.26)	0.86 (0.22)	0.88 (0.26)	0.139	0.475	0.497
	Stride Length (m)	0.82 (0.21)	0.86 (0.21)	0.86 (0.18)	0.87 (0.19)	0.017*	0.609	0.128
	Stride Time (s)	1.07 (0.15)	1.06 (0.12)	1.03 (0.10)	1.03 (0.14)	0.814	0.346	0.613

Values are mean ± SD. * Indicates significant main effect, *p* < 0.05. 2MWT, two minute walk test. 10MWT, ten meter walk test. Asterisks and bold font indicate significance.

Among the 10MWT conditions, we observed one group x time interaction, which was for stride time in the 10MWT at a comfortable pace. Pairwise comparisons revealed that ballet participants reduced stride time (*p* = 0.029) whereas wellness participants increased it (*p* = 0.008). Additionally, for the main effect of time, stride length in the backward direction increased regardless of group.

### 3.2 Gait variability results

For gait variability in the 2MWT, there was a main effect of time showing an increase (i.e., worsening) in gait velocity variability (Table 4). An interaction effect showed that this was driven by the ballet group.

**Table 4:** Gait variabilities.

		**Ballet**		**Wellness**				
		Pre	Post	Pre	Post	Main effect of time	Main effect of group	Interaction effect
2 MWT	Velocity CV	3.84 (1.09)	5.03 (2.3)	4.45 (1.95)	4.51 (1.53)	0.015*	0.913	0.028*
	Stride length CV	2.82 (0.88)	3.17 (1.40)	3.10 (1.39)	3.21 (1.19)	0.069	0.654	0.356
	Stride time CV	1.98 (0.71)	2.65 (1.9)	2.38 (1.06)	2.38 (0.92)	0.094	0.851	0.099
	10 MWT comfortable	Velocity CV	4.13 (1.42)	3.62 (1.36)	3.69 (2.01)	3.50 (1.09)	0.101	0.488	0.444
Stride length CV		3.20 (1.00)	2.94 (1.26)	2.66 (1.33)	2.68 (0.70)	0.396	0.183	0.328
Stride time CV		1.96 (0.69)	1.82 (0.55)	1.20 (1.12)	1.86 (0.57)	0.266	0.914	0.910
10 MWT dual task		Velocity CV	5.22 (2.56)	4.42 (1.62)	4.03 (1.27)	4.62 (1.76)	0.685	0.320	0.013*
	Stride length CV	4.17 (1.88)	3.48 (1.28)	3.14 (0.98)	3.64 (1.47)	0.667	0.254	0.011*
	Stride time CV	2.65 (1.31)	2.43 (0.89)	2.17 (0.65)	2.37 (0.89)	0.918	0.298	0.109
	10 MWT fast as possible	Velocity CV	3.93 (1.21)	4.18 (1.21)	3.44 (1.06)	4.00 (1.69)	0.070	0.322	0.481
Stride length CV		3.30 (1.13)	3.58 (1.48)	2.87 (1.06)	2.87 (0.87)	0.482	0.056	0.476
Stride time CV		2.05 (0.64)	2.23 (0.80)	2.17 (0.74)	2.09 (0.40)	0.676	0.953	0.235
10 MWT backward		Velocity CV	17.68 (1.09)	13.50 (1.23)	13.58 (1.04)	13.52 (1.18)	<0.001*	0.185	0.001*
	Stride length CV	13.92 (0.81)	11.14 (1.05)	11.05 (0.78)	10.72 (1.0)	0.016*	0.157	0.054
	Stride time CV	6.13 (0.46)	5.26 (0.46)	6.34 (0.44)	6.07 (0.44)	0.025*	0.394	0.229

Values are mean ± SD. * Indicates significant main effect, *p* < 0.05. 2MWT, two minute walk test. 10MWT, ten meter walk test. CV, coefficient of variation. Asterisks and bold font indicate significance.

However, in the 10MWT, the ballet group showed the most improvement in the DT and backwards conditions. In the DT condition, there were group x time interactions for velocity CV and stride length CV revealing that ballet participants improved whereas wellness participants worsened. In the backward direction, main effects of time showed that both groups improved variability measures, including velocity CV, stride length CV, and stride time CV. There were no main effects of group, but a significant group x time interaction for velocity CV indicated that this improvement was driven by the ballet group.

### 3.3 Balance results

**Figure 1:**
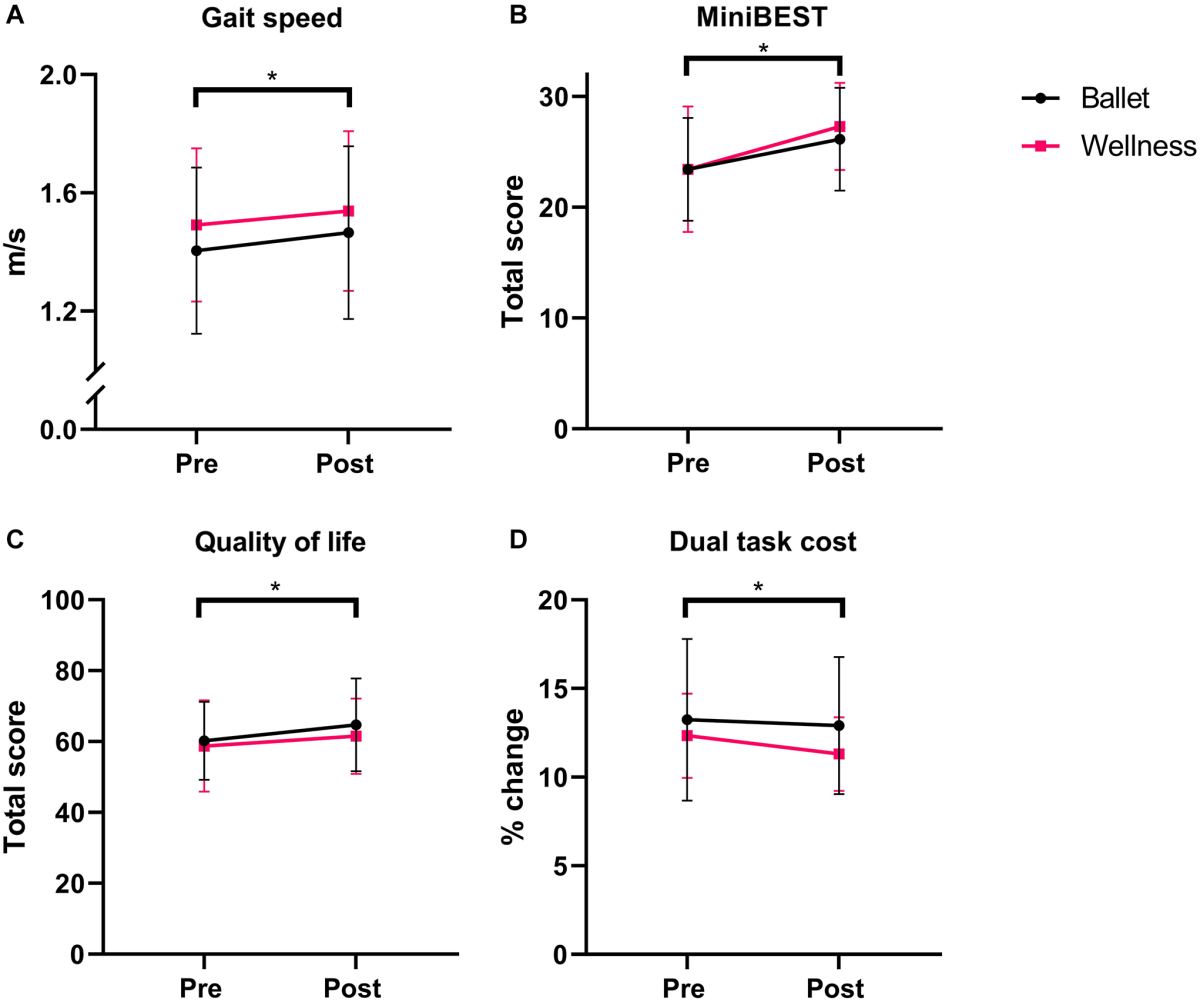
Key results pre- to post-intervention between groups. **(A)** Gait speed in 2MWT. **(B)** Mini-BESTest. **(C)** SF-36. **(D)** Dual Task Cost. * Indicates significance at *p* < 0.05.

There was a main effect of time for scores on the Mini-BESTest (*F*_1,42_ = 38.15, *p* < 0.001) (Figure 1) and balance confidence as measured by the ABC (*F*_1,42_ = 10.69, *p* = 0.002). The ballet group increased Mini-BEST scores from a mean (range) of 24 (13,32) to 28 (18,32) and the wellness group increased 24 (10,31) to 28 (20,32). For balance confidence measured via the ABC, there was a main effect of time The group × time interaction trended toward significance (*F*_2,41_ = 3.86, *p* = 0.056) as the ballet group increased ABC scores from 86.57 (2.4) to 92.64 (1.57) while the wellness group increased from 89.99 (2.31) to 91.50 (1.50).

Static balance also exhibited a main effect of time as measured by postural sway during single leg stance. From pretest to posttest, the duration that participants were able to hold a static standing posture in single leg stance (*F*_1,42_ = 12.69, *p* < 0.001) increased [Ballet: 12.48 s (8.45) to 15.85 s (8.53), Wellness: 14.20s (8.49) to 16.22 s (9.19)]. In single leg stance, RMS Acc (*F*_1,42_ = 5.11, *p* = 0.029) decreased over time, indicating more stability. There were no main effects of group.

There was no change in the TUG duration (*p* = 0.066), but there was a main effect of time for the DT-TUG duration (*F*_1,42_ = 4.279, *p* = 0.045), with the ballet group duration reducing from 14.23 s (±4.56) to 13.90s (±3.87) and the wellness group reducing from 13.33 s (±2.37) to 12.30s (±2.07). Dual task cost decreased from pretest to posttest (*F*_1,42_ = 4.744, *p* = 0.035) with no effect of group.

### 3.4 Fall rates

There was a significant effect of group on fall rates [χ^2^(1) = 5.096, *p* = 0.024], as ballet participants reduced fall rates from pretest to posttest (19 to 0%) whereas wellness participants increased fall rates (9–13%). When removing the covariate adjustment for participants’ baseline fall rates, a significant difference in posttest falls remains [χ^2^(1) = 4.092, *p* = 0.043].

### 3.5 Quality of life, mood, and class satisfaction

Both classes were associated with improvements in quality of life as measured by the SF-36 (*F*_1,42_ = 7.66, *p* = 0.008). Mood, as measured by the GDS, did not change significantly in either group (*F*_1,42_ = 3.45, *p* = 0.07) [Ballet: 5.29 (±0.21) to 4.95 (±0.19); Wellness: 5.17 (±0.20) to 4.96 (±0.182)].

The ballet group responded more positively in the post-intervention survey, with 71% of participants wishing to continue the classes compared to 47% of the wellness participants. Ballet participants reported that the classes improved strength, balance, and flexibility, that they were beneficial for daily activities, and that the classes made them “feel different,” “want to move again,” and inspired a “positive vibe.” Wellness participants gave feedback that they thought the classes were effective, enjoyable, and educational. Many noted a benefit of connecting with other women during a time of isolation due to COVID-19. Others noted that they became more grateful for their situations and learned to appreciate their health and communities.

## 4 Discussion

This study is among the first to explore the potential of virtual dance classes to benefit multiple domains of health. Our results show that both virtual ballet and wellness classes improved gait velocity, postural sway, and quality of life for older women. The ballet participants, however, improved more on complex, challenging tasks, such as dual-task walking and backward walking. Ballet classes seemed to be engaging and enjoyable, allowing people to creatively and artistically express agency and to connect kinesthetically with other women during a time of isolation. Wellness classes provided an outlet for informed knowledge-sharing while bonding with women at similar stages of life experience. The parallel improvements in quality of life and mobility highlight the importance of considering social engagement during both virtual and in-person movement therapies.

Ballet participants exhibited two notable improvements in gait variability, a known marker of fall risk, both in the most complex and attention-demanding walking paradigms: dual-task walking and backward walking. Dance is known to improve dual tasking, particularly in people with basal ganglia disorders like Parkinson disease (Kalyani et al., 2019). Ballet requires constant task-switching as people aim to remember combinations, integrate visual and auditory cues, perform complex movement patterns, and fulfill aesthetic goals. Significant reductions in variability in dual task and backward walking may reflect the potential of dance—and ballet, specifically—to train attention and focus during complex motor tasks, freeing up cognitive reserves for more successfully fulfilling the motor task. Such benefits may hold therapeutic meaning as challenging gait tasks require more cognitive reserve and frequently pose the greatest risk of falls (Verghese et al., 2002; Springer et al., 2006; Bayot et al., 2020). This explanation would support a recent study of young adult ballet dancers showing that ballet skills transfer to complex walking tasks such as walking across a narrow beam (Sawers and Ting, 2015).

In another complex motor task, the DT-TUG, which has been shown to discriminate fallers from non-fallers (Schoene et al., 2013), our participants’ (regardless of group) mean duration at baseline (13.78 s) surpasses a previously validated threshold of 13.5 s and suggests that many were within the range of fall risk (Steffen et al., 2002). The mean reduction to 13.06 s at posttest suggests that, overall, participants reduced indicators of fall risk. The improvement for both groups in dual task cost suggests that the interventions may have improved cognitive flexibility and adaptability during complex motor tasks, which could help people maintain postural control during tasks that divide attention and elicit falls (Verghese et al., 2002).

In terms of balance, improvements in the Mini-BESTest and ABC suggest global improvements in both objective and subjective stability. We expected ballet participants to improve both balance and balance confidence, confirming our hypotheses. However, wellness participants also improved in multiple domains of balance as well, which was unexpected. As the Mini-BESTest is negatively associated with fall prediction, the mean improvements for the ballet group of 2.4 and the wellness group of 3.1 are meaningful. A 1-point reduction in this test can increase the odds of a fall in the next 6 months by 14% for adults over the age of 60 and this percentage increases each decade (Magnani et al., 2020). Hence, our participants who improved their scores may have significantly reduced their risk of falls.

Static balance improved in only a few measures. Improvement in SLS duration and RMS acceleration are important as they correlate to reduced fall risk (Alqahtani et al., 2017; Omaña et al., 2021). Surprisingly, ballet participants had no better outcomes on static balance than wellness participants; however, this does confirm past findings that in-person ballet classes did not significantly alter postural stability during static poses (Weighart and DiPasquale, 2020). In previous work, this was attributed to an over-reliance on holding onto a ballet barre during class, which may not sufficiently challenge proprioceptive systems and therefore not alter performance on free-standing balance tasks (Weighart and DiPasquale, 2020). As we did not control for holding onto equipment, this may explain our similar results.

The ballet intervention successfully reduced falls while the wellness intervention did not. This corroborates past evidence that group exercise programs reduce falls whereas knowledge-based, educational interventions designed to reduce falls do not (Gillespie et al., 2021). The reduction of falls among ballet participants suggests that even small improvements in various domains of mobility and postural control may make a difference in everyday fall risk.

Both groups improved quality of life and spoke positively about the opportunity for virtual connection during a time of social isolation. As conversation time was built into the control intervention, wellness participants spent more time getting to know one another than ballet participants and may have developed bonds over shared experiences related to aging and health. Since the wellness class often showed parallel benefits to the ballet class, it is possible that the social outlet afforded during the wellness classes led to psycho-emotional benefits that translated to a global effect on motor outcomes.

Qualitatively, ballet participants reported higher class satisfaction and a stronger desire for the course to continue compared to the wellness participants. Their positive feedback emphasized the highly motivational nature of dance interventions, which may elicit deeper engagement than standard exercise (Earhart, 2009; Hwang and Braun, 2015). Participants in the ballet group also emphasized the importance of connecting creatively with other women, which highlights the interconnectedness of psycho-social–emotional wellbeing, artistic expression, and mobility (Kyrdalen et al., 2019). Lastly, they indicated that increasing mobility through engaging, expressive means gave them a sense of agency over both their physical and mental health. Such comments parallel a recent report that suggested that “the joyful, social, creative and expressive elements of dance are perhaps the precise reason for its efficacy within health contexts” (Introducing “Dance for Health”, 2020). Taken together, the success of the ballet intervention from the participants’ viewpoint further highlights the importance of considering psychosocial benefits of group movement-based interventions.

Several limitations to this study should be noted when interpreting the results. The high dropout rate reduced our sample size. The positive outcomes in the exit questionnaires could have been biased because they reflected the views of those who remained in the study. We chose the wellness class as a control intervention to provide similar social and attentional interactions but without a movement component. The control intervention did not involve movement so we cannot know how ballet classes would compare to other forms of exercise. Future studies could compare the ballet intervention to different movement-based interventions, but that was not the goal of this initial study. Lastly, some of the significant changes we saw were small and may not be clinically relevant.

## 5 Conclusion

This study is among the first to explore the effects of virtual ballet as a therapeutic intervention for older women. The results highlight the importance of social interaction as a key component when developing future interventions to target physical, mental, and psychosocial wellbeing in older adults, as well as the enjoyability of dance relative to other approaches. Future work should compare the effects of virtual and in-person movement classes, while accounting for the interaction between multiple domains related to brain and body health.

## Data availability statement

The raw data supporting the conclusions of this article will be made available by the authors, without undue reservation.

## Ethics statement

The studies involving humans were approved by Washington University School of Medicine Institutional Review Board. The studies were conducted in accordance with the local legislation and institutional requirements. The participants provided their written informed consent to participate in this study.

## Author contributions

EH: Conceptualization, Data curation, Formal analysis, Funding acquisition, Investigation, Methodology, Project administration, Resources, Supervision, Validation, Visualization, Writing – original draft, Writing – review & editing. AH: Investigation, Writing – review & editing. LT: Investigation, Writing – review & editing. SB: Investigation, Writing – review & editing. GE: Conceptualization, Funding acquisition, Investigation, Methodology, Writing – review & editing.
